# Underuse of Postoperative Radiation After Nipple-Sparing Mastectomy for Standard Radiation Indications

**DOI:** 10.1016/j.adro.2024.101569

**Published:** 2024-07-14

**Authors:** Wesley J. Talcott, Gustavo N. Marta, Meena S. Moran

**Affiliations:** a Department of Radiation Medicine, Northwell Health, New York; b Department of Radiation Oncology, Hospital Sírio-Libanês, Brazil; c Latin American Cooperative Oncology Group, Brazil; d Department of Therapeutic Radiology, Yale University School of Medicine, New Haven, Connecticut

## Abstract

**Purpose:**

Nipple areola complex-sparing surgeries, such as nipple-sparing mastectomy (NSM), are increasingly used for women with early-stage breast cancer. In the postoperative setting, 2 major indications for postoperative radiation (PORT) with/without regional nodal irradiation (RNI) are: positive margins (margin+) and pathologically involved lymph nodes (pN+). The frequency of these adverse pathologic features and the rate of PORT utilization following NSM for these 2 indications are unknown. We determined the frequency of margin+ and pN+ following NSM compared with nipple-sparing lumpectomy/breast-conserving surgery [BCS] and identified trends in appropriate PORT administration for these standard indications in the NSM setting.

**Methods and Materials:**

Using the National Cancer Database (NCDB), women diagnosed with cT1 to cT3,N0M0 invasive carcinoma between 2004 and 2017 who underwent NSM were compared with those who underwent BCS (with nipple preservation). The frequencies of margin+ and pN+ by surgical subtype and use of PORT with/without RNI were assessed by cohort to determine if the type of surgery was associated with radiation delivery. Overall survival between the 2 cohorts was also compared. We performed univariable/multivariable logistic and Cox regression with ORs to control for confounders.

**Results:**

Of 624,075 women included, 611,907 underwent BCS, and 12,168 underwent NSM. The surgical margin+ rate was significantly higher for NSM at 4.5% (n = 544) than for BCS at 3.7% (n = 22,449) (*P* < .001) and remained significant on multivariable analysis (MVA; OR, 1.13; CI, 1.03-1.25; *P* = .012). Use of PORT for margins+ was significantly lower by MVA after NSM (OR, 0.07; CI, 0.06-0.09; *P* < .001). Similarly, pN+ rate was significantly higher for NSM at 22.5% (n = 2740) versus BCS at 13.5% (n = 82,288) (*P* < .001), retaining significance on MVA (OR, 1.12; CI, 1.06-1.19; *P* < .001). For pN+ undergoing NSM, PORT with RNI was delivered significantly less often on MVA (OR, 0.73; CI, 0.67-0.81; *P* < .001). Neither high-risk subgroup had differences in overall survival on MVA.

**Conclusions:**

NSM is associated with a higher rate of margin+ and pN+ compared with BCS. Radiation is underused after NSM for these standard indications. Our results highlight the need to further refine patient selection for NSM and the importance of communicating the higher potential for adverse pathologic features (and thus, the potential need for radiation) to patients undergoing NSM.

## Introduction

For women with early-stage breast cancer, preservation of the nipple-areolar complex (NAC) and skin flap overlying the glandular breast tissue results in better patient-reported cosmetic and functional outcomes than if these structures are sacrificed.^1^ As a result, surgical techniques that spare the NAC have increased in use.^2^ Sparing of the NAC is generally very common after a breast conservation procedure because it aligns with the surgical foundation of preserving the affected breast while optimizing cosmesis (eg, lumpectomy/breast-conserving surgery [BCS]).^3^^,^^4^ Similarly, in the mastectomy setting, the use of nipple-sparing techniques (eg, nipple-sparing mastectomy [NSM]) offers the patient a better chance for preserving body image and nipple function, thus enhancing quality of life.^1^^,^^5^

The indications for radiation after definitive surgery with either BCS or mastectomy are well delineated.^6^ For example, most patients undergoing a breast conservation approach receive radiation therapy to either the whole breast (with or without regional nodal irradiation [RNI]) or around the lumpectomy region alone (accelerated partial breast irradiation) to reduce local recurrence in the residual breast tissue.^7^, ^8^, ^9^, ^10^ In contrast, after mastectomy, the presumption is that the at-risk breast tissue has been removed. Thus, most patients do not require adjuvant radiation, with postoperative radiation therapy (PORT +/− RNI) typically reserved for patients with higher-risk features such as surgical margin positivity, pathologic lymph node involvement, or large tumor size (>5 cm).^11^^,^^12^ However, the oncologic safety of NSM is still a matter of debate, and when extrapolating indications for PORT after simple/modified radical mastectomy to the NSM setting, there are neither prospective published data nor consensus statements/guidelines to facilitate decision making.^13^, ^14^, ^15^ Given the association between PORT and potential postoperative complications in NSM patients,^16^^,^^17^ particularly NAC necrosis or compromised cosmetic outcome, reluctance for PORT delivery for patients undergoing NSM would be understandable. . We hypothesize that PORT is underused in the NSM setting, and to better understand these patterns of care, we used the National Cancer Database (NCDB) to investigate 2 standard indications for PORT (positive margin [margin+] status and pathologic node positivity[pN+]) in early-stage breast cancer patients undergoing NSM. We compared a larger cohort of NSM patients with BCS patients to assess whether the use of PORT for these 2 indications was equivalent.

## Methods and Materials

We used the 2020 release of the NCDB to assess patterns of care among patients with early-stage, clinically node-negative breast cancer. A joint project of the American Cancer Society and the Commission on Cancer of the American College of Surgeons, the NCDB, is a nationwide, facility-based, comprehensive clinical surveillance resource oncology data set that currently captures 72% of all newly diagnosed malignancies in the United States annually.^18^ Patients without prior cancer history were included if they had a preoperative biopsy histologically confirming breast cancer diagnosed with cT1 to cT3,cN0M0 invasive ductal or lobular carcinoma between the years 2004 and 2017; all patients who underwent definitive NSM or BCS resection (with exclusion of those patients who had nipple-areolar removal with local excision) with known final margin status were included. A margin+ was defined as gross or microscopic tumor present at the final surgical margin, including re-resection margins.

Descriptive statistics with χ^2^ testing, univariable analysis and multivariable analysis (MVA) using logistic regression with OR, and multivariable Cox proportion hazards regression analysis (with 95% CI) were performed using Stata software (v13.1) to determine final margin+ or pathologic node involvement (pN+) rates for each surgical cohort (NSM vs BCS) to determine whether PORT +/− RNI receipt varied by type of surgery. Overall survival (OS) for patients with margin+ or pN+ was analyzed as a function of surgical procedure performed to determine if there were any long-term survival differences. To control for observable biases between treatment cohorts, regressions incorporating disease and patient characteristics hypothesized to contribute to margin positivity, nodal positivity, adjuvant radiation delivery, and survival were performed. These variables included age, year of diagnosis, comorbidity burden (Charlson-Deyo comorbidity index), race/ethnicity (White non-Hispanic, Black, White Hispanic, and other), insurance status, academic versus nonacademic treatment center, median annual income in a patient's area of residence (≥$63,000 and <$63,000), distance from a treatment center (≥20 miles and <20 miles), clinical tumor size, neoadjuvant systemic therapy, histologic grade, ER, PR, human epidermal growth factor receptor 2 status, and genomic risk score (high risk, low risk, and not performed). For adjuvant radiation therapy delivery and survival, variables for pathologic tumor size and nodal stage, extent of lymph node evaluation, and adjuvant therapy receipt were also included in regression models. All clinical-pathologic parameters that met a significance threshold of *P* < .10 on univariable logistic regression were included in the respective multivariable model.

An additional sensitivity analysis was performed for patients who received diagnoses after January 1, 2015, in order to determine if the publication of 2 large randomized phase 3 trials (both accruing majority pN+ patients and demonstrating a disease-free survival benefit associated with adjuvant radiation delivery)^19^^,^^20^ affected patterns of postoperative radiation delivery. The study was granted exemption by the institutional review board.

## Results

**Figure 1:**
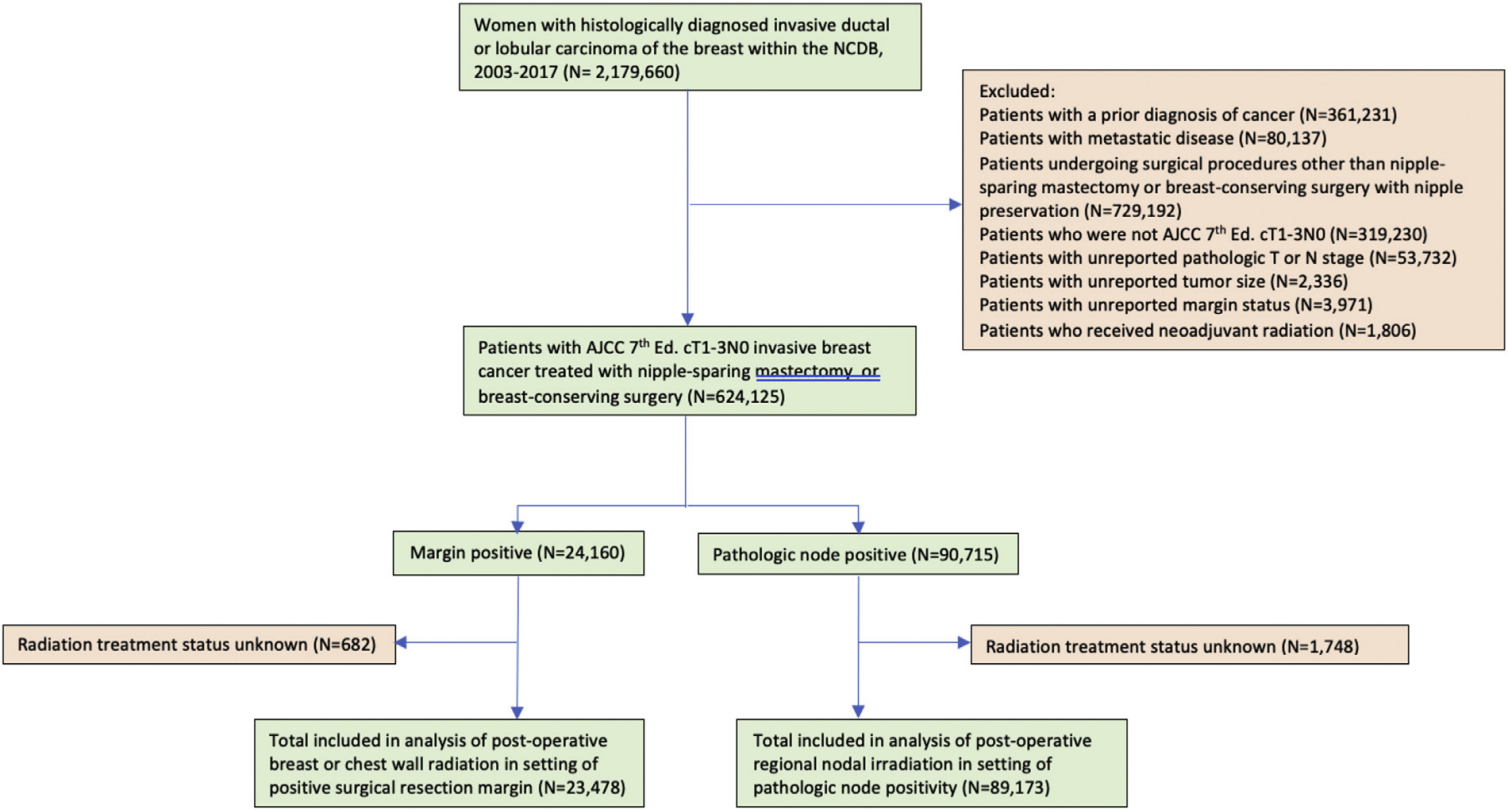
Selection map for inclusion. *Abbreviation:* NCDB = National Cancer Database.

A total of 624,075 women were assessed; most of them underwent BCS (n = 611,907). NSM was performed in 12,168 patients (Fig. 1). Patients undergoing NSM were younger (median age, 50 years [IQR, 44-58 years] for NSM versus 63 years [IQR, 54-71 years] for BCS; *P* < .001) (Table 1). The median length of time from diagnosis to definitive surgery was significantly longer for patients undergoing NSM (52 days, IQR, 36-80 days) versus BCS (31 days, IQR, 20-47 days) (*P* < .001). There were statistically significant differences in the clinical T stage at presentation between the 2 surgical cohorts: patients presented with cT1 in 66.8% versus 81.8%, cT2 in 29.9% versus 17.5%, and cT3 in 3.4% vs 0.7% for NSM and BCS, respectively (*P* < .001). Moreover, the percentage of patients receiving neoadjuvant chemotherapy was significantly different between NSM and BCS (8.1% vs 2.5%, *P* < .001). Additional differences were found in the NSM versus BCS cohorts for the delivery of adjuvant hormone therapy, 78.1% versus 74.1% (*P* < .001), and adjuvant systemic chemotherapy, 33.7% versus 24.4% (*P* < .001), respectively. However, the overall receipt of PORT was significantly lower after NSM, 17.2% versus 83.3%, for BCS (*P* < .001). Documented reasons for omission of PORT differed significantly between the 2 groups in the setting of both margin+ (*P* < .001) and pN+ (*P* < .001) (Table 2).

**Table 1:** Cohort characteristics of patients receiving BCS and NSM

Variable	Variable subcategory	BCS	NSM
Age at diagnosis (y)	-	63 (IQR, 54-71)	50 (IQR, 44-58)
Year of diagnosis	2004-2010	184,735 (30.2)	570 (4.7)
	2011-2017	427,172 (69.8)	11,598 (95.3)
Pathologic tumor size	≤0.5 cm	59,854 (9.8)	1054 (8.7)
	>0.5 to ≤1.0 cm	157,793 (25.8)	2025 (16.6)
	>1.0 to ≤2.0 cm	264,607 (43.2)	4675 (38.4)
	>2 to ≤5.0 cm	124,456 (20.3)	3895 (32.0)
	>5 to ≤10.0 cm	5197 (0.9)	519 (4.3)
Clinical tumor stage	cT1	500,723 (81.8)	8123 (66.7)
	cT2	107,019 (17.5)	3633 (29.8)
	cT3	4,165 (0.7)	412 (3.4)
Pathologic nodal stage	pNX	17,697 (2.9)	36 (0.3)
	pN0	512,071 (83.7)	9402 (77.3)
	pN1	73,677 (12.0)	2349 (19.3)
	pN2	6699 (1.1)	310 (2.6)
	pN3	1763 (0.3)	71 (0.6)
Surgical assessment of nodes	Nodal aspiration only	1553 (0.4)	58 (0.0)
	Sentinel node biopsy	285,486 (46.7)	7,807 (64.1)
	Axillary node dissection	69,872 (11.4)	3166 (26.0)
	No regional nodal assessment	30,796 (5.0)	141 (1.0)
	Nodal assessment performed but unspecified	224,102 (36.6)	1012 (8.3)
	Regional nodal assessment unknown	131 (0.0)	0 (0.0)
Hormone receptor status	ER+/PR+	467,350 (76.4)	9668 (79.3)
	ER+/PR-	60,077 (9.9)	1007 (8.3)
	ER−/PR+	4634 (0.1)	93 (0.1)
	ER–/PR–	73,797 (12.9)	1416 (11.6)
	Receptor status unknown	5107 (0.0)	
Her2 status	Her2+	45,591 (7.5)	1520 (12.5)
	Her2–	425,626 (69.6)	10,221 (83.9)
	Her2 unknown	140,724 (23.0)	443 (3.6)
Grade	1	170,171 (27.8)	2653 (21.8)
	2	272,548 (44.5)	5738 (47.1)
	3	145,113 (23.7)	3303 (27.1)
	Grade unknown	24,109 (3.9)	490 (4.0)
Lymphovascular invasion	Negative	367,395 (77.3)	8648 (72.7)
	Positive	52,189 (11.0)	2083 (17.5)
	Unknown	55,726 (11.7)	1168 (9.8)
Race/ethnicity	White non-Hispanic	478,555 (78.2)	9405 (77.2)
	Black	58,665 (9.6)	891 (7.3)
	White Hispanic	25,059 (4.1)	715 (5.9)
	Other	49,662 (8.1)	1173 (9.6)
Charlson-Deyo comorbidity index score	0	515,649 (84.3)	11,072 (90.9)
	1	76,133 (12.4)	939 (7.7)
	2	14,782 (2.4)	125 (1.0)
	≥3	5377 (0.9)	48 (0.4)
Distance from a radiation center	<20 miles	429,184 (70.1)	7604 (62.4)
	≥20 miles	182,757 (29.9)	4580 (37.6)
Genomic risk score	Not high risk	132,906 (21.7)	4286 (35.2)
	High risk	28,213 (4.6)	830 (6.8)
	Not performed	450,822 (73.7)	7068 (58.0)
Treatment center type	Academic	179,200 (29.3)	4317 (35.4)
	Nonacademic	418,816 (68.4)	6247 (51.3)
	Unspecified/other	13,925 (2.3)	1620 (13.3)
Insurance type	Private	317,056 (51.8)	9734 (79.9)
	Nonprivate	294,885 (48.2)	2450 (20.1)
Median household income in patient's area code	>$63,000	233,190 (42.7)	5845 (55.7)
	≤$63,000	312,525 (57.3)	4648 (44.3)
Final surgical margins	Negative	589,442 (96.3)	11,640 (95.5)
	Positive	22,499 (3.7)	544 (4.5)
Pathologic node positivity	Negative	529,570 (86.6)	9444 (77.5)
	Positive	82,288 (13.5)	2740 (22.5)
Postoperative radiation therapy to the breast or chest wall	No	93,554 (15.7)	10,069 (82.0)
	Yes	518,387 (84.7)	2189 (18.0)
Regional nodal irradiation	No	566,501 (92.6)	10,970 (90.0)
	Yes	45,440 (7.4)	1214 (10.0)
Neoadjuvant chemotherapy	No	596,849 (97.5)	11,186 (91.9)
	Yes	15,058 (2.5)	982 (8.1)
Adjuvant chemotherapy	No	453,355 (74.1)	9504 (78.1)
	Yes	158,552 (25.9)	2664 (21.9)
Hormone therapy	No	149,464 (24.4)	4059 (33.4)
	Yes	462,443 (75.6)	8109 (66.6)
Total	-	611,907	12,168

*Abbreviations:* BCS = breast-conserving surgery; ER = estrogen receptor; HER-2 = human epidermal growth factor receptor 2; NSM = nipple-sparing mastectomy; PR = progesterone receptor.

**Table 2:** Reason for no PORT in the margin positive and pathologic node-positive setting. “Not part of the first line of therapy” indicates that multiple options were offered and the patient selected treatment that did not include radiation of the primary site, or if the option of “no treatment” was accepted by the patient

Reason for no adjuvant radiation	Margin positive		Node positive	
	NSM	BCS	NSM	BCS
Not part of the first line of therapy	86.8%	60.3%	82.3%	54.6%
Contraindicated because of other patient risk factors	2.6%	7.8%	1.7%	6.3%
Recommended but refused by the patient	7.9%	28.2%	14.6%	35.1%
Recommended but not delivered for an unknown reason	2.6%	3.8%	1.4%	4.1%

*Abbreviations:* BCS = breast-conserving surgery; NSM = nipple-sparing mastectomy; PORT = postoperative radiation therapy.

The overall rate of final margin+ across all patients included in this analysis was 3.7% (n = 23,033). On univariable analysis, the margin+ rate was significantly higher for the NSM cohort compared with BCS (4.5% vs 3.7%, *P* < .001) and remained significant on MVA (OR, 1.15; CI, 1.04-1.27; *P* = .005) (Table 3). Furthermore, among the patients with a final margin+ and documented PORT delivery status, PORT was found to be administered significantly less often for NSM-margin+ patients than for BCS-margin+ patients (48.2% vs 79.2%, *P* < .001) and remained significant on MVA (OR, 0.09; CI, 0.07-0.11; *P* < .001) (Table 4). Even among the subset of patients with the smallest (cT1) tumors, NSM (compared with BCS) was associated with a higher margin+ rate (OR, 1.33; CI, 1.19-1.48; *P* < .001) and lower PORT use rate in the setting of margin+ (OR, 0.05; CI, 0.04-0.07; *P* < .001).

**Table 3:** Multivariate analysis for margin positivity

Variable		OR	95% CI	*P* value
Surgery type	BCS	Ref		
	NSM	1.15	1.04-1.27	.005
Year of diagnosis (per year after 2004)	-	0.97	0.96-0.98	<.001
Age at diagnosis (per year)	Age	1.00	1.00-1.01	<.001
Race/ethnicity	White non-Hispanic	Ref		
	Black	1.19	1.14-1.25	<.001
	White Hispanic	1.19	1.11-1.27	<.001
	Other/NOS	1.04	0.99-1.09	.165
Median income in patient zip code	≥$63,000	Ref		
	<$63,000	0.95	0.93-0.98	.001
Distance from radiation treatment center	<20 miles	Ref		
	≥20 miles	0.98	0.94-1.01	.18
Charlson-Deyo comorbidity index score	0	Ref		
	1	0.93	0.89-0.97	.001
	2	0.92	0.84-1.01	.076
	≥3	1.09	0.95-1.25	.198
Pathologic tumor size	≤1 cm	Ref		
	1-2 cm	1.32	1.28-1.37	<.001
	2-3 cm	1.79	1.71-1.87	<.001
	3-4 cm	2.27	2.14-2.42	<.001
	4-5 cm	2.74	2.50-3.00	<.001
	5-10 cm	4.62	4.23-5.06	<.001
Pathologic nodal stage	pN0	Ref		
	pN1	1.42	1.37-1.48	<.001
	pN2	1.89	1.72-2.09	<.001
	pN3	2.71	2.32-3.17	<.001
	unknown	1.16	1.07-1.26	<.001
Histologic grade	1	Ref		
	2	1.20	1.16-1.24	<.001
	3	1.10	1.05-1.15	<.001
	Unknown	1.33	1.24-1.43	<.001
Estrogen receptor positivity	ER+	1.22	1.16-1.28	<.001
Her2 receptor status	Negative	Ref		
	Positive	1.19	1.12-1.25	<.001
	Unknown	1.07	1.02-1.12	.009
Nodal assessment performed	Sentinel node biopsy	Ref		
	Axillary node dissection	1.04	1.00-1.08	.049
	Aspiration of node only	1.49	1.17-1.91	.001
	No regional node assessment	2.76	2.58-2.95	<.001

*Abbreviations:* BCS = breast-conserving surgery; ER = estrogen receptor; Her2 = human epidermal growth factor receptor 2; NSM = nipple-sparing mastectomy.

**Table 4:** Multivariate analysis for receipt of postoperation radiation therapy in the setting of margin positivity

Variable		OR	95% CI	*P* value
Surgery type	BCS	Ref		
	NSM	0.09	0.07-0.11	<.001
Year of diagnosis (per year after 2004)	-	0.98	0.97-0.99	<.001
Age at diagnosis (per year)	Age	0.97	0.97-0.98	<.001
Center type	Academic	Ref		
	Nonacademic	1.14	1.05-1.24	.003
Race/ethnicity	White non-Hispanic	Ref		
	Black	0.79	0.70-0.90	<.001
	White Hispanic	0.87	0.72-1.05	.139
	Other/NOS	0.94	0.81-1.08	.374
Charlson-Deyo comorbidity index score	0	Ref		
	1	0.87	0.78-0.98	.018
	2	0.87	0.69-1.08	.207
	≥3	0.86	0.61-1.20	.378
Median income in patient zip code	≥$63,000	Ref		
	<$63,000	1.02	0.94-1.10	.689
Distance from radiation treatment center	<20 miles	Ref		
	≥20 miles	0.91	0.83-1.01	.072
Insurance type	Private	Ref		
	Nonprivate	0.87	0.79-0.96	.007
Pathologic tumor size	≤1 cm	Ref		
	1-2 cm	0.68	0.61-0.75	<.001
	2-3 cm	0.63	0.56-0.71	<.001
	3-4 cm	0.54	0.46-0.64	<.001
	4-5 cm	0.39	0.31-0.49	<.001
	5-10 cm	0.57	0.45-0.72	<.001
Pathologic nodal stage	pN0	Ref		
	pN1	0.89	0.79-1.00	.044
	pN2	0.60	0.47-0.78	<.001
	pN3	0.67	0.45-1.02	.06
	unknown	0.17	0.15-0.19	<.001
Lymphovascular invasion	-	1.02	0.90-1.16	.798
Histologic grade	1	Ref		
	2	0.97	0.88-1.07	.562
	3	0.98	0.86-1.11	.746
	Unknown	0.96	0.79-1.17	.692
Progesterone receptor positivity	-	0.65	0.58-0.72	<.001
Genomic risk score	Low risk	Ref		
	High Risk	0.58	0.45-0.76	<.001
	No risk score performed	0.48	0.42-0.55	<.001
Adjuvant chemotherapy	-	2.74	2.44-3.09	<.001
Hormone therapy	-	5.29	4.84-5.78	<.001
Neoadjuvant chemotherapy	-	2.04	1.57-2.64	<.001

*Abbreviations:* BCS = breast-conserving surgery; NSM = nipple-sparing mastectomy.

The overall rate of pN+ across all patients included in this analysis was 13.6% (N = 85,028). The rate of pN+ disease was significantly higher after NSM (n = 2740) than after BCS (n = 82,288) at 22.5% versus 13.5% (*P* < .001), respectively. These findings remained significant on MVA when accounting for confounding factors, demonstrating higher pathologic nodal involvement after NSM than after BCS (OR, 1.08; CI, 1.03-1.15; *P* < .001) (Table 5). Furthermore, MVA of receipt of PORT + RNI for pN+ demonstrated that NSM patients with pN+ were significantly less likely to receive PORT + RNI than BCS patients (OR, 0.73; CI 0.66-0.81; *P* < .001) (Table 6). When analyzing only macroscopic nodal metastases for the pN+ (excluding micrometastases[pN1mic]), patients undergoing NSM remained less likely to receive PORT + RNI on MVA compared with those undergoing BCS (OR, 0.83; CI, 0.75-0.93; *P* = .001).

**Table 5:** Multivariate analysis for node positivity

Variable		OR	95% CI	*P* value
Surgery type	BCS	Ref		
	NSM	1.08	1.03-1.15	<.001
Year of diagnosis (per year after 2004)	-	1.02	1.02-1.02	<.001
Age at diagnosis (per year)	Age	0.99	0.99-0.99	<.001
Center type	Academic	Ref		
	Nonacademic	0.98	0.96-1.00	.03
Race/ethnicity	White non-Hispanic	Ref		
	Black	1.03	1.00-1.06	.02
	White Hispanic	1.03	0.99-1.08	.10
	Other/NOS	0.91	0.88-0.94	<.001
Charlson-Deyo comorbidity index score	0	Ref		
	1	1.07	1.04-1.10	<.001
	2	1.12	1.07-1.19	<.001
	≥3	1.17	1.07-1.28	<.001
Distance from radiation treatment center	<20 miles	Ref		
	≥20 miles	1.07	1.05-1.09	<.001
Insurance type	Private	Ref		
	Nonprivate	1.00	0.98-1.02	.67
Clinical T stage	cT1	Ref		
	cT2	1.02	0.99-1.04	.16
	cT3	0.97	0.88-1.07	.50
Pathologic tumor size	≤1 cm	Ref		
	1-2 cm	2.89	2.83-2.96	<.001
	2-3 cm	4.69	4.54-4.83	<.001
	3-4 cm	5.54	5.31-5.79	<.001
	4-5 cm	5.73	5.38-6.11	<.001
	5-10 cm	6.04	5.56-6.57	<.001
Lymphovascular invasion	-	5.02	4.91-5.13	<.001
Estrogen receptor positivity	-	1.72	1.66-1.78	<.001
Progesterone receptor positivity	-	1.20	1.16-1.23	<.001
Genomic risk score	Low risk	Ref		<.001
	High risk	0.67	0.64-0.70	<.001
	No risk score performed	1.30	1.27-1.33	<.001
Nodal assessment performed	Sentinel node biopsy	Ref		<.001
	Axillary node dissection	4.79	4.71-4.87	<.001
	Aspiration of node only	1.10	0.92-1.31	.30
	No regional node assessment	0.07	0.06-0.08	<.001
Neoadjuvant chemotherapy	-	0.84	0.80-0.88	<.001
Hormone therapy	-	1.72	1.66-1.79	<.001

*Abbreviations:* BCS = breast-conserving surgery; NSM = nipple-sparing mastectomy.

**Table 6:** Multivariate analysis for regional nodal irradiation in the setting of node positivity

Variable		OR	95% CI	*P* value
Surgery type	BCS	Ref		
	NSM	0.73	0.66-0.81	<.001
Year of diagnosis (per year after 2004)	-	1.08	1.07-1.08	<.001
Age at diagnosis (per year)	Age	0.99	0.99-1.00	<.001
Center type	Academic	Ref		
	Nonacademic	1.09	1.05-1.12	<.001
Race/ethnicity	White non-Hispanic			
	Black	0.95	0.90-1.00	0.063
	White Hispanic	0.87	0.81-0.94	<0.001
	Other/NOS	0.96	0.90-1.01	.133
Median income in patient zip code	≥$63,000	Ref		
	<$63,000	1.08	1.04-1.11	<.001
Pathologic tumor size	≤1 cm	Ref		
	1-2 cm	1.08	1.03-1.13	0.003
	2-3 cm	1.17	1.11-1.24	<.001
	3-4 cm	1.23	1.14-1.32	<.001
	4-5 cm	1.28	1.15-1.43	<.001
	5-10 cm	1.51	1.34-1.71	<.001
Estrogen receptor positivity	-	0.61	0.56-0.66	<.001
Histologic grade	1	Ref		
	2	1.06	1.02-1.11	.005
	3	1.09	1.03-1.15	.001
	Unknown	1.03	0.93-1.13	.573
Lymphovascular invasion	-	1.12	1.08-1.16	<.001
Genomic risk score	Low risk	Ref		
	High risk	0.85	0.78-0.92	<.001
	No risk score performed	1.00	0.96-1.05	.955
Neoadjuvant chemotherapy	-	1.35	1.24-1.48	<.001
Hormone therapy	-	2.10	1.97-2.24	<.001
Adjuvant chemotherapy	-	1.37	1.32-1.42	<.001

*Abbreviations:* BCS = breast-conserving surgery; NSM = nipple-sparing mastectomy.

When assessing only patients who received diagnoses after January 1, 2015, there was a significant increase in PORT use after NSM for patients with pN+, with 34.6% of patients who received diagnoses before this date receiving RNI (N = 339), compared with 41.9% of those who received diagnoses after this date (N = 693)(*P* < .001). Despite this increase, on MVA, NSM continued to be associated with a lower likelihood of receiving RNI versus BCS among cases diagnosed from 2015 onward (OR, 0.70; CI, 0.62-0.79; *P* < .001) (Table E1).

For patients with positive surgical margins (n = 20,588) at a median follow-up of 62.2 months, the OS outcomes did not differ significantly between NSM and BCS (0.62; CI, 0.30-1.31; *P* = .21). Similarly, for pN+ patients (n = 75,166), at median follow-up of 60.5 months, there was no survival difference between NSM and BCS (OR, 1.01; CI 0.80-1.28; *P* = .93).

## Discussion

In this study, we have used the NCDB to analyze the use of PORT+/–RNI in the setting of NSM for 2 standard indications for postoperative radiation (margin+ and pathologic node positivity). We chose to compare NSM with BCS (instead of simple or modified radical mastectomy) to reduce the selection bias inherent to this population of patients based on the following: (1) our assumption that patients undergoing NSM would be a selected group of early-stage, young(er), and lower-risk cohort and would be less likely to have confounding factors for omission of PORT (ie, multiple comorbidities or elderly age); (2) the early-stage, breast conservation patients with nipple-sparing would be more similar to the NSM cohort than simple mastectomy cohort; and (3) our exploratory analysis (not shown but available) confirmed that the use of mastectomy as a comparison arm with NSM had a higher degree of variability in age and significantly more high-risk features than the comparison of NSM with BCS. Our findings suggest that NSM is associated with higher rates of final surgical margin+ and pN+ disease compared with BCS. Furthermore, patients undergoing NSM were found to have received PORT+/–RNI significantly less often than BCS for the standard indications of margin+ and macroscopic node-positive disease.

It was notable that the final margin+ rates were higher among patients who underwent NSM than BCS. We acknowledge that this finding is surprising on its surface given that NSM necessitates excision of more breast tissue than BCS. One explanation may be that patients undergoing NSM had larger tumors; however, our subset analysis of even the cohort with the smallest tumor size (cT1) suggests that the margin+ rate is higher in the NSM cohort than in the BCS cohort. This finding is likely due to the relative increase in difficulty in performing a re-excision after a margin+ following NSM, when immediate reconstruction is performed, in comparison with a margin+ after BCS, which generally has a localizable tumor bed with potential for additional tissue to be removed. Alternatively, this finding may be due to reluctance perform a nipple-sacrificing technique in the setting of NAC involvement, given the time and energy invested in the nipple-sparing procedure. Finally, the lack of clearcut guidelines delineating the clinical scenarios when NSM is contraindicated may result in surgeons offering/performing NSM more often for patients with higher risks of margin+, with less referrals for PORT. Similarly, the rate of pN+ disease was significantly higher in the NSM compared with the BCS cohort, and among these pN+, NSM patients were found to be less likely to receive PORT + RNI. Although future data, potential biologic assays or genomic risk scores offer promise to identify subsets of pN+ cohorts in whom PORT can be safely omitted , at present, this practice would be in opposition to guideline recommendations on appropriate adjuvant therapy. Together, these findings suggest that the patient selection for NSM needs additional refinement. Furthermore, our findings suggest that current standard indications/guidelines for PORT are not currently being appropriately utilized after NSM. The underuse of PORT was also evident in the subset of NSM patients with gross macrometastasis (micrometastatic disease excluded) and, remarkably, was also underused in the clinical situation where axillary dissection was omitted in NSM patients with macrometastatic disease on sentinel node biopsy alone. This underutilization of PORT+/–RNI, despite clearcut indications (whether intentional or unintentional), may be due to concerns for cosmetic complications including NAC loss/necrosis or reluctance of surgeons or patients to pursue additional local-regional treatment after such an extensive procedure as NSM, with the perception of low risk of recurrence. Therefore, our findings highlight the need to further refine patient selection for NSM so that margin+ rates and node positivity in patients undergoing NSM are reduced, thus reducing the need for PORT +/− RNI. It is also important to re-emphasize to patients considering treatment options that (nipple-sparing) lumpectomy and radiation provide similar/superior cosmetic and functional outcomes^21^ as NSM without any differences in survival, ultimately allowing for an overall lower treatment burden. Lastly, it is critical that surgeons inform patients being considered for NSM that, given the significantly higher likelihood of positive margins and potential for pathologic nodal involvement, PORT +/− RNI may be indicated in the postoperative setting. Patient presentation at multidisciplinary tumor boards with radiation oncologists, medical oncologists, and surgical oncologists may increase both proper patient selection for NSM and the proper use of PORT following NSM.

We performed an exploratory analysis of patients who received diagnoses and treated after the publication of 2 pivotal radiation trials in 2015 assessing the benefit of PORT in patients and further established the benefits of PORT in the pN+ setting. Our assessment indicated that there has indeed been an increase in the uptake of PORT use after NSM for ypN+ patient since 2015. Despite this increase, our multivariate analysis suggested that NSM remains associated with a lower likelihood of receiving RNI compared with BCS among cases diagnosed after 2015. Thus, our findings suggest that even in a more contemporary patient subset, there appears to remain patterns of underutilization of PORT in pN+ patients following NSM.

Our study has several limitations. The NCDB does not capture data on local-regional recurrence or cause-specific mortality. Although generally, the primary benefit of PORT +/− RNI delivery for margin+ or pN+ disease is diminishing local-regional relapse, it is important to note that PORT+/–RNI has, in fact, been demonstrated to decrease distant recurrence and result in a small, but statistically significant, breast cancer-specific survival benefit.^7^^,^^22^ Thus, underuse of PORT for these clinical scenarios that have the potential for residual microscopic disease after NSM may have important implications on outcomes that cannot be measured by this data set. Although it is reassuring that the underuse of PORT does not appear to result in significant treatment-related differences or compromise OS, it is likely that most recurrences experienced in these cohorts were local-regional in nature and theoretically may be salvaged with additional surgery, radiation, and systemic therapy, hence ultimately resulting in similar long-term survival outcomes. Furthermore, any differences in survival outcomes from radiation are typically not apparent until 15 years or more after treatment, and given the relatively short median follow-up of this cohort, we would not anticipate being able to demonstrate differences in survival outcomes based on the benefits of radiation alone. The NCDB does not capture all pathologic details about a tumor specimen. Margin status is reported as negative, microscopically positive, or macroscopically positive; whether a microscopic margin was focally, extensively, or multifocally positive is not reported. Although current guidelines strongly recommend radiation in the setting of any margin+, whether physicians felt more comfortable omitting radiation in the focally margin+ setting cannot be determined. Lastly, it is noteworthy that the most common reason for omission of PORT among margin- or node-positive NSM patients was that “radiation was not felt to be indicated” (by the provider) and, in fact, the proportion of patients refusing PORT or with contraindications for PORT was greater for BCS than for NSM in these settings, further suggesting that underuse of radiation following NSM is more a function of provider preference than patient-related factors.

## Conclusions

In summary, our analysis of PORT use following NSM for 2 commonly accepted standard indications (margin positivity and pathologic node positivity) suggests that PORT is being underused compared with BCS. Although it is understandable that providers may be less likely to refer an NSM patient for radiation because of the higher possible risk of NAC necrosis and adverse functional or cosmetic outcomes associated with radiation, the higher rates of margin+ and pN+ disease without PORT +/− RNI delivery is alarming. Although this data set is limited in its inability to analyze local-regional recurrence outcomes and potential differences in long-term survival outcomes, our results highlight the need to further refine patient selection for NSM. Furthermore, for those pursuing NSM, the comparable survival outcomes of NSM and BCS in early-stage breast cancer, and the higher potential for adverse pathologic features requiring PORT should be discussed with patients, so informed discussions are made and local-regional outcomes are not ultimately compromised.

## Disclosures

Dr. Meena Moran is Vice Chair of the National Comprehensive Cancer Network (NCCN) Breast Panel.

## Appendix Supplementary materials

Supplementary materials Image, application 1

## Footnotes

Supplementary material associated with this article can be found in the online version at doi:10.1016/j.adro.2024.101569.
