# Supplementary material for: Underuse of Postoperative Radiation After Nipple-Sparing Mastectomy for Standard Radiation Indications
Source: Adv Radiat Oncol. 2024 Jul 14;9(9):101569. doi: 10.1016/j.adro.2024.101569 (PMC11345291; doi:10.1016/j.adro.2024.101569)
Supplement: Supplementary materials [file mmc1.docx]

Supplementary Table 1 – Multivariate analysis for regional nodal irradiation in setting of node positivity for patients diagnosed after January 1^st^, 2015.

|  | | | | | |
| --- | --- | --- | --- | --- | --- |
| Variable | | OR | 95% CI | | p value |
| Surgery type | BCS | Ref |  |  |  |
|  | NSM | 0.70 | 0.62 | 0.79 | <0.001 |
| Year of diagnosis (per year after 2004) |  | 1.06 | 1.03 | 1.10 | <0.001 |
| Age at diagnosis (per year) | Age | 0.99 | 0.99 | 1.00 | <0.001 |
| Center type | Academic | Ref |  |  |  |
|  | Non-academic | 1.10 | 1.04 | 1.17 | 0.001 |
| Race/Ethnicity | White non-Hispanic |  |  |  |  |
|  | Black | 0.97 | 0.88 | 1.06 | 0.456 |
|  | White Hispanic | 0.80 | 0.71 | 0.91 | <0.001 |
|  | Other/NOS | 0.90 | 0.81 | 1.00 | 0.041 |
| Median income in patient zip code | ≥$63,000 | Ref |  |  |  |
|  | <$63,000 | 1.02 | 0.97 | 1.09 | 0.317 |
| Pathologic tumor size | ≤1cm | Ref |  |  |  |
|  | 1-2cm | 1.13 | 1.04 | 1.23 | 0.004 |
|  | 2-3cm | 1.23 | 1.12 | 1.34 | <0.001 |
|  | 3-4cm | 1.24 | 1.10 | 1.39 | 0.001 |
|  | 4-5cm | 1.36 | 1.14 | 1.62 | 0.001 |
|  | 5-10cm | 1.58 | 1.31 | 1.91 | <0.001 |
| Pathologic nodal stage | pN1  pN2  pN3  Unknown | Ref  2.00  2.17  0.90 | 1.74  1.72  0.75 | 2.20  2.74  10.9 | <0.001  <0.001  0.936 |
| Estrogen receptor positivity |  | 0.52 | 0.45 | 0.61 | <0.001 |
| Histologic grade | 1 | Ref |  |  |  |
|  | 2 | 1.08 | 1.00 | 1.16 | 0.005 |
|  | 3 | 1.04 | 0.95 | 1.13 | 0.001 |
|  | Unknown | 1.00 | 0.81 | 1.24 | 0.573 |
| Lymphovascular invasion |  | 1.13 | 1.06 | 1.19 | <0.001 |
| Her2 receptor Status | Her2- | Ref |  |  |  |
|  | Her2+ | 1.26 | 1.14 | 1.39 | <0.001 |
|  | Her2 status unknown | 0.90 | 0.51 | 1.59 | 0.716 |
| Nodal assessment performed | Sentinel node biopsy  Axillary dissection  Nodal aspiration  No nodal assessment | Ref  0.98  0.99  1.01 | 0.93  0.61  0.71 | 1.04  1.63  1.44 | 0.567  0.974  0.934 |
| Neoadjuvant chemotherapy |  | 1.38 | 1.20 | 1.61 | <0.001 |
| Endocrine therapy |  | 2.71 | 2.40 | 3.04 | <0.001 |
| Adjuvant chemotherapy |  | 1.61 | 1.52 | 1.71 | <0.001 |

Note: NSM = nipple-sparing mastectomy; BCS = nipple-sparing breast-conserving surgery.
